# Multi-Stacked Supported Lipid Bilayer Micropatterning through Polymer Stencil Lift-Off

**DOI:** 10.3390/membranes5030385

**Published:** 2015-08-28

**Authors:** Yujie Zhu, Ahmed Negmi, Jose Moran-Mirabal

**Affiliations:** Department of Chemistry and Chemical Biology, McMaster Univerisity, 1280 Main Street West, Hamilton, Ontario L8S 4M8, Canada

**Keywords:** stacked supported lipid bilayer, phase separation, micropatterning, polymer stencil lift-off, fluorescence microscopy

## Abstract

Complex multi-lamellar structures play a critical role in biological systems, where they are present as lamellar bodies, and as part of biological assemblies that control energy transduction processes. Multi-lamellar lipid layers not only provide interesting systems for fundamental research on membrane structure and bilayer-associated polypeptides, but can also serve as components in bioinspired materials or devices. Although the ability to pattern stacked lipid bilayers at the micron scale is of importance for these purposes, limited work has been done in developing such patterning techniques. Here, we present a simple and direct approach to pattern stacked supported lipid bilayers (SLBs) using polymer stencil lift-off and the electrostatic interactions between cationic and anionic lipids. Both homogeneous and phase-segregated stacked SLB patterns were produced, demonstrating that the stacked lipid bilayers retain lateral diffusivity. We demonstrate patterned SLB stacks of up to four bilayers, where fluorescence resonance energy transfer (FRET) and quenching was used to probe the interactions between lipid bilayers. Furthermore, the study of lipid phase behaviour showed that gel phase domains align between adjacent layers. The proposed stacked SLB pattern platform provides a robust model for studying lipid behaviour with a controlled number of bilayers, and an attractive means towards building functional bioinspired materials or devices.

## 1. Introduction

In living systems, multilamellar membranous structures are present as lamellar bodies and as key elements in many active biological assemblies that control energy transduction processes [1]. *In vitro* models of lipid multilayers that can mimic these natural systems are important tools for biophysical studies of membranes. Such stacked lipid bilayer systems have been previously used to study interactions between neighbouring bilayers, such as membrane fusion [2] and anomalous swelling [3], as well as the structure of membranes as they interact with polypeptides [4]. Stacked bilayers are also of interest for practical applications such as cell sensing [5], drug delivery [6] and disease diagnosis [7]. For example, it has been recently reported that stacked SLBs can be used as a tunable substrate to study dynamic, mechano-regulated cell linkages and cellular mechano-sensing [5,8]; therefore, methods for reconstructing lipid multilayer structures at the nano- or microscale *in vitro* need to be developed for better mimicking the complexity of natural biomembranes.

A number of strategies have been developed for building stacked SLBs from the bottom-up *in vitro*. Electrostatic interactions between cationic and anionic lipid bilayers [9] and between phospholipids and silica templates [10] have been used to form double stacked bilayers and highly ordered hybrid multilamellar assemblies respectively. In addition, specific biological interactions involving biotin-streptavidin coupling [11] and DNA hybridization [12], as well as covalent bonds involving inter-bilayer maleimide-thiol coupling [13], have been used to form double bilayer systems with variable stability. Despite the efforts made in building stacked bilayers, to date there are no reported techniques that can easily pattern them at the micron scale and in large array format. A key limitation for the *in situ* patterning of stacked lipid bilayers is the need to conduct the formation of the bilayer stack and the selective patterning under aqueous conditions. Thus, the majority of techniques reported have been limited to patterning of a single SLB. Microcontact printing has been used for patterning multiple lipid bilayers, but with poor control of lipid organization and stack thickness [14]. Similarly, capillary assembly and dip-pen nanolithography (DPN) and have been successfully used to pattern multi-stacked SLB through the use of microstructured surfaces [15] and by direct writing of arbitrary patterns [16,17], respectively. Moreover, a method combining microcontact printing, nanoimprint lithography, and dip-pen nanolithography has been developed to create nanostructured lipid multilayer arrays [18]. However, these techniques are resource intensive, are not able to control the number of bilayers accurately or have difficulty in patterning phase-segregated SLBs. Therefore, new techniques that allow simple patterning of lipid multilayers in large areas with accurate stack thickness control and which can pattern phase segregated bilayers are needed.

Polymer stencil lift-off (PSLO) is a robust technique that uses a microfabricated Parylene stencil [19] to pattern a wide range of biomaterials, such as proteins [20], DNA [21], cellulose fibrils [22], or cells [23], at the micron scale (its patterning resolution only limited by the lithographic technique used to define the stencil openings). The PSLO technique has also been successfully applied in functional SLB patterning [24], which allows faithful transfer of uniform SLB patterns in aqueous condition with sub-micron resolution [25]. Here, we report the extension of the PSLO technique as a simple and direct approach to pattern a controlled number of stacked SLBs under aqueous conditions. SLB stacks were formed by electrostatic interactions between cationic and anionic lipids. This strategy allows patterning large areas of SLB stacks of micrometer dimensions, with accurately controlled number of layers and compositions. Both homogeneous and phase-segregated stacked SLB patterns were achieved. To our knowledge, this is the first report of patterned SLB stacks that are built from the bottom-up with controlled distributions of lipids, which opens up possibilities for new biophysical studies on interactions between membranes and between membranes and membrane associated proteins, as well as future applications in cell culture and drug screening.

## 2. Results and Discussion

### 2.1. Supported Lipid Bilayer Micropatterning

**Figure 1:**
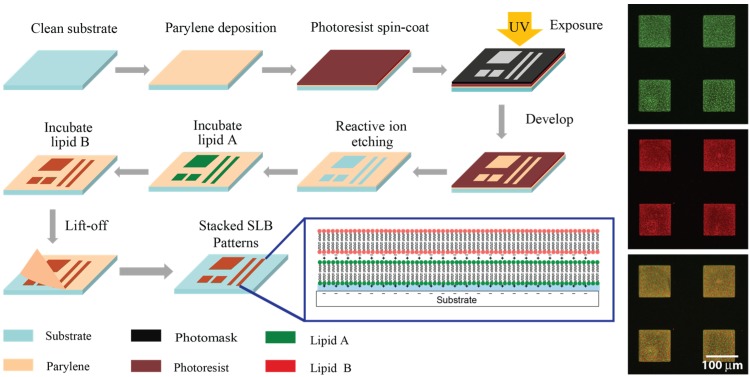
Illustration of the PSLO patterning process for stacked SLBs. Parylene film is deposited onto a clean substrate, and photoresist is patterned using photolithography. After reactive ion etching of Parylene and removal of photoresist, small unilamellar lipid vesicles are deposited on the stencils and incubated to form SLBs. The final step is to lift off the stencil, revealing stacked SLB patterns. Inset: side view illustration of a double lipid bilayer with cationic and anionic lipids, and fluorescence images of a two-bilayer stack.

Supported Lipid Bilayer stacks were deposited into micron-sized patterns through the use of Polymer Stencil Lift-Off (PSLO, Figure 1). In this process, Parylene was first deposited through chemical vapour deposition onto SiO_2_ surfaces to form a 500–1000 nm thick, conformal, pinhole-free film. Parylene films were then patterned thorough standard photolithography and reactive ion etching processes. This resulted in polymer stencils with arrays of features with 2–200 μm critical dimensions, which were subsequently cleaned with acetone, UV-O_3_, and 100 mM sodium hydroxide solution to remove any photoresist and polymer residue in the opening areas, producing smooth surfaces suitable for SLB formation through vesicle fusion. By incubating small unilamellar lipid vesicles (SUVs) on the substrates under the appropriate temperature, SLBs formed within the Parylene stencil openings. For single lipid bilayer patterning, the stencil could be removed after the first incubation step, while for multiple SLB stack formation the stencil remained in place until after the last bilayer was deposited. The Parylene stencil served as a stable physical barrier preventing the SLBs from spreading and remained intact during the whole SLB stacking process, which was conducted under aqueous conditions. The deposited lipids formed micron-sized single and multiple SLBs domains, demonstrating the suitability of PSLO for patterning stacked SLBs.

### 2.2. Homogeneous SLB Stacks

**Figure 2:**
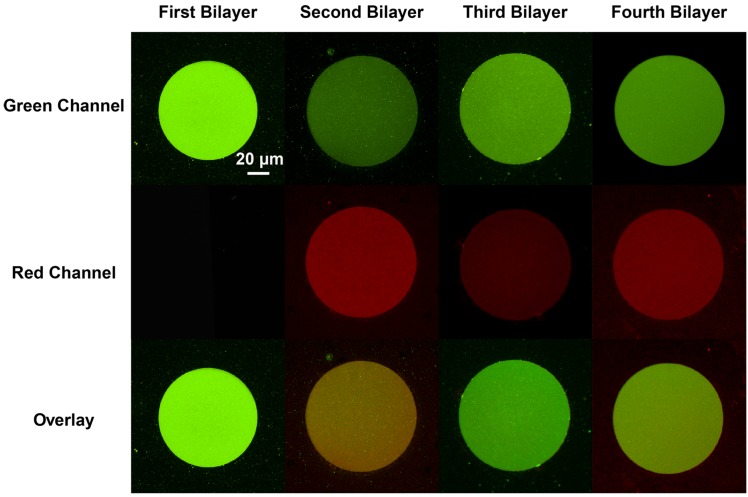
Epifluorescence images of four-bilayer homogeneous stacked SLB micropatterns. The first and third bilayers were composed of DOPC:DOTAP:DHPE-OG and can be observed in green channel. Second and fourth bilayers were composed of DOPC:DMPS:DHPE-LR, and can be observed in red channel (middle). The bottom row presents an overlay of both red and green channels. All images were acquired at the same magnification.

**Figure 3:**
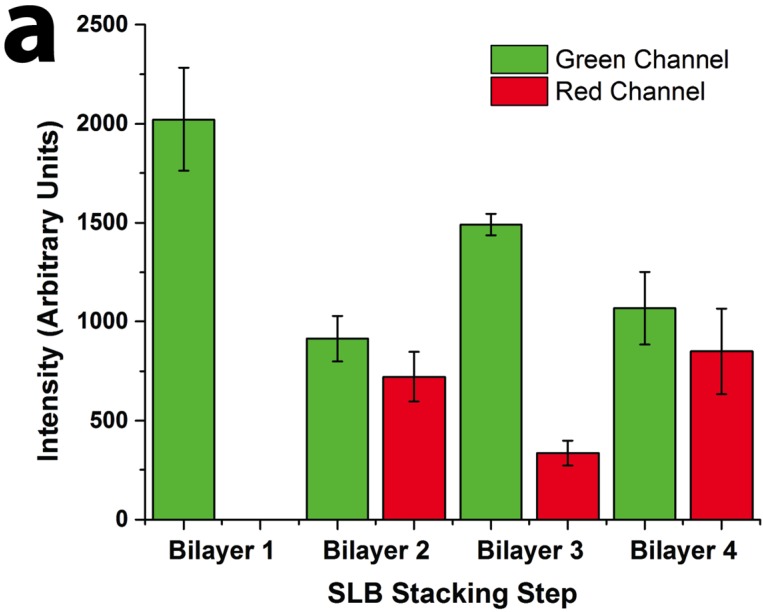

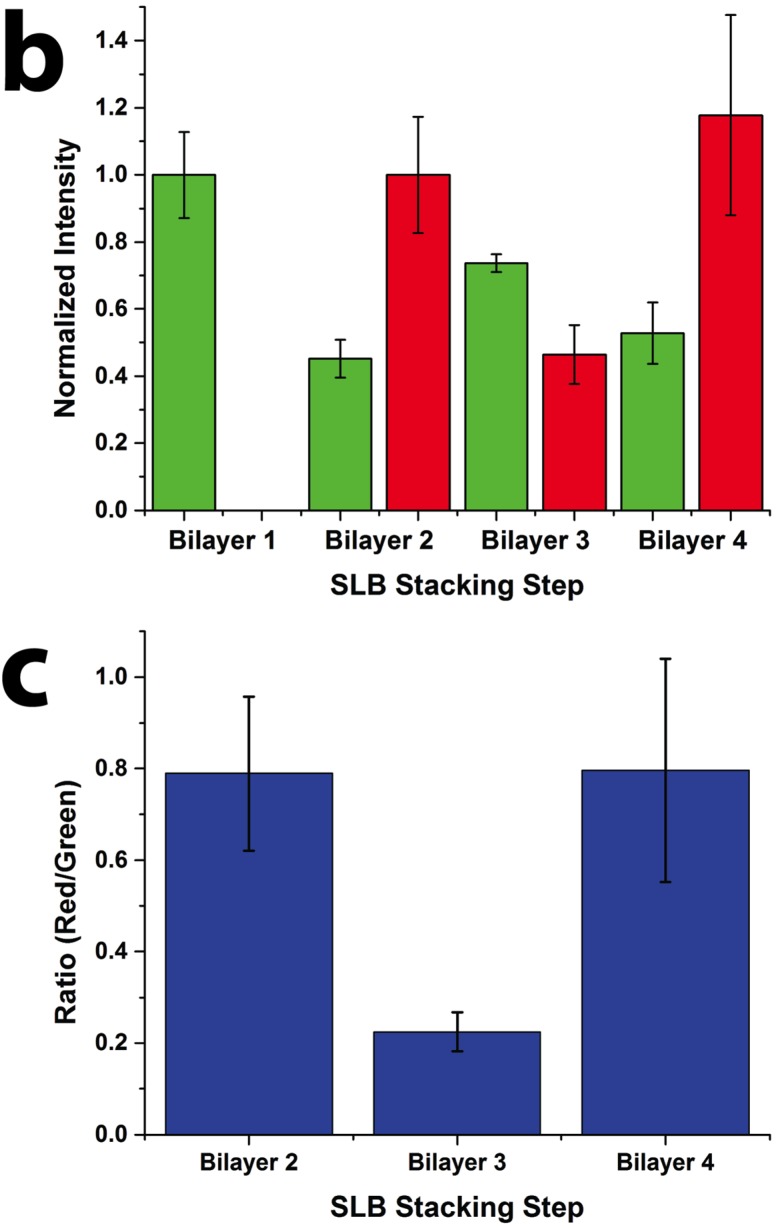
Quantitative fluorescence intensity analysis of stacked SLB formation. (**a**) Raw fluorescence intensity of green (DHPE-OG) and red (DHPE-LR) channels of the four-bilayer SLB patterns after each stacking step; (**b**) normalized intensity of each stacking step (green channel normalized to bilayer 1 and red channel normalized to bilayer 2 values); and (**c**) ratio of the raw intensities of red to green channels at each stacking step.

The ability to pattern SLB stacks through the PSLO technique was first tested using lipid mixtures that formed homogenous bilayers. Two kinds of 1,2-dioleoyl-sn-glycero-3-phosphocholine (DOPC) vesicle solutions were prepared: one was doped with 10% cationic lipid 1,2-dioleoyl-3-trimethylammonium-propane (DOTAP), and the other with 10% anionic lipid 1,2-dimyristoyl-sn-glycero-3-phospho-L-serine (DMPS). As the SiO_2_ substrate was negatively charged under slightly basic buffer conditions, a positively-charged SLB containing DOTAP was formed first by vesicle fusion directly on the substrate. With the first cationic bilayer formed, the anionic lipid vesicles were then incubated to form the second SLB. The electrostatic interactions between the oppositely-charged lipids promoted the fusion of anionic vesicles on top of the underlying cationic bilayer. By alternating the charge of the subsequent layers, three- and four-bilayer stacks could be similarly formed. We demonstrated the formation of up to four homogenous SLBs with DOPC as the main component through this bilayer-by-bilayer approach. However, it must be noted that any number of bilayers is possible provided the thickness of the resulting stack does not exceed the stencil thickness. The stacked SLB patterns were visualized by fluorescence microscopy during the stack formation (Figure 2). Oregon Green labeled 1,2-dihexadecanoyl-sn-glycero-3-phosphoethanolamine (DHPE-OG) and Lissamine Rhodamine B 1,2-dihexadecanoyl-sn-glycero-3-phosphoethanolamine (DHPE-LR) were added to the vesicles at low concentrations (0.1% molar ratio) to label the SLBs, and were imaged in the green and red fluorescence channels, respectively. The images show that fluorescence intensity of both channels changed with the addition of each new bilayer. Specifically, after the second bilayer (labeled with DHPE-LR) and third bilayers were formed, the intensity of DHPE-OG in the first bilayer and the DHPE-LR in the second bilayer greatly decreased, a change ascribed to fluorescence resonance energy transfer (FRET) or collisional quenching mechanisms, respectively. On one hand, in FRET, Oregon Green, as a donor chromophore, initially in its electronic excited state, transferred energy through non-radiative dipole–dipole coupling to the acceptor chromophore, Lissamine Rhodamine (LR), resulting in decreased green channel intensity; on the other hand, the addition of a third bilayer and the close proximity of DHPE-OG molecules to DHPE-LR could cause collisional quenching, leading to reduced intensities in the red channel, too. Quantitative fluorescence analysis was conducted by measuring the absolute and relative fluorescence intensities of each bilayer (relative intensity normalized to the intensity of the first bilayer added in each channel), as shown in Figure 3. The ratio of red over the green channel raw intensities (Figure 3c) increases with the deposition of LR labeled SLBs, and decreases with the addition of OG-labeled SLBs. The trend for fluorescence intensity change was in good agreement with expected intensity variations based on the successive addition of fluorescent bilayers, taking into account FRET and quenching effects, further confirming the formation of patterned SLB stacks. In addition, fluorescence recovery after photobleaching (FRAP) experiments were performed on each successive bilayer added (Figure S1), where the observation of homogeneous recovery indicated that each bilayer of the stack retained good mobility at room temperature. The fluorescence background observed outside the patterns in bilayers 2 and 3 of Figure 3 was generated by vesicles adhered on the Parylene stencil, as the stencil was not lifted off until all four bilayers had been deposited. The fluorescence images, fluorescence intensity analysis, and FRAP tests demonstrated that fully-mobile SLB stacks could be formed by layer-by layer deposition and easily patterned using the PSLO approach.

### 2.3. Stacked SLBs Containing Phase-Segregated Domains

**Figure 4:**
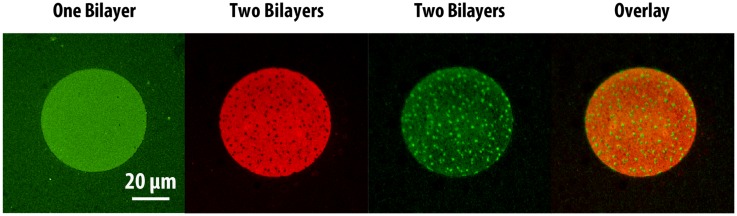
Epifluorescence images showing patterns of two-bilayer SLB stacks consisting of a homogeneous bottom bilayer and a phase-segregated top bilayer. The first lipid bilayer (composed of DOPC: DOTAP: DHPE-OG) is observed in the green channel, while the second lipid bilayer (composed of DOPC: DSPC: DOPS: DHPE-LR) can be observed in the red channel. The formation of the second bilayer leads to significant fluorescence intensity changes in the first bilayer. The overlaid image of the two bilayers shows the fluorescence patterns are complimentary, due to FRET.

The same bilayer-by-bilayer deposition and PSLO approach was applied to create stacked SLB patterns containing phase-segregating lipid compositions. The anionic and cationic lipids used in these experiments (DOPS, DOTAP) partition preferentially into the liquid-disordered phase due to their unsaturated carbon chains, which result in lower transition temperatures (*T*_m_ = −11 °C and −11.9 °C, respectively). Phase segregation was achieved by introducing 1,2-distearoyl-sn-glycero-3-phosphocholine (DSPC, a fully saturated lipid with *T*_m_ = 55 °C) into the DOPC:DOPS/DOTAP:DHPE-OG/LR lipid mixtures. Homogeneous, fully-mixed bilayers were successfully formed at 60°C (above the DSPC transition temperature), after which cooling to room temperature (21 °C) resulted in the formation of visible phase-segregated lipid domains. Since the DHPE-OG/LR dyes preferentially partition into the liquid-disordered phase (primarily composed of DOPC), the gel phase consisting mostly of DSPC would be observed as dark domains. First, we demonstrated the ability to form two-bilayer stacks composed of a phase-segregated bilayer on top of a homogeneous one (Figure 4). As expected, the first lipid bilayer deposited, with composition DOPC:DOTAP:DHPE-OG, was uniformly fluorescent in the green channel. However, upon formation of a second phase-segregated SLB, with composition DOPC:DSPC:DOPS:DHPE-LR, it was observed that the green channel now showed a complementary pattern to the red channel, as shown in the overlay image of Figure 4. This phenomenon can be readily explained by the close proximity and interaction between the fluorescent probes in each of the bilayers within the stack, which results in FRET. Since FRET could only occur between DHPE-OG molecules (green) in the first bilayer (homogeneous) that came into close proximity with DHPE-LR molecules (red) in the second bilayer (phase-segregated), and given that the DHPE-LR molecules partitioned preferentially to the fluid phase, areas of the first bilayer overlapping the gel domains in the second bilayer appeared brighter than the surrounding areas. This phenomenon directly probes the interactions between the two bilayers, and demonstrates that the stacked bilayers are in close proximity. Additionally, the observation of phase segregation in the second bilayer, coupled with FRAP measurements on both homogenous and phase-segregated layers in this experiment (Figure S2), confirmed the formation of fully-mobile heterogeneous SLB stacks through the PSLO technique.

**Figure 5:**
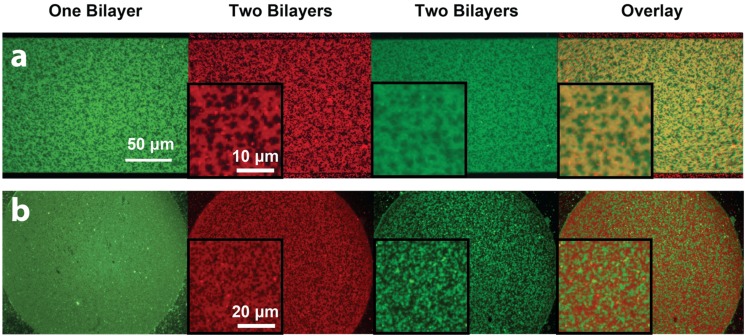
Epifluorescence images of stacked phase-segregated SLB patterns. The first lipid bilayer was composed of DOPC:DSPC:DOTAP:DHPE-OG (green channel), while the second lipid bilayer was composed of DOPC:DSPC:DOPS:DHPE-LR (red channel). Insets show zoomed-in areas of the patterned bilayers. (**a**) When the first bilayer showed good phase segregation (green), the second lipid bilayer would phase segregate in the same pattern (red). The fluorescence intensity of first bilayer would decrease due to FRET, and the overlaid image of the two channels showed alignment of gel phase domains in the two bilayers; (**b**) When the first bilayer did not phase-segregate, the second SLB would still show gel-phase domains. The first bilayer appeared dimmer in areas that overlapped with the liquid disordered phase of the second bilayer. Image contrast has been adjusted to facilitate the visualization of the overlapping patterns in the stacked lipid bilayers.

Next, we explored the possibility of patterning two-bilayer stacks where both bilayers would phase-segregate, and investigated the phase behaviour of the stacked bilayers. Small unilamellar lipid vesicles composed of DOPC:DSPC:DOTAP:DHPE-OG were deposited and incubated at 60 °C to form the first bilayer. It was observed that when the substrate was properly cleaned (*i.e.*, the bilayer mobility was appropriate), micron-scale gel domains formed on the cationic bilayer upon slow cooling. As expected, the DHPE-OG molecules preferentially partitioned into the liquid disordered phase, leaving the gel phase dark (Figure 5a, green channel). A second anionic phase-segregated bilayer (with composition DOPC:DSPC:DOPS:DHPE-LR, Figure 5a red channel) could then be formed on top by incubating the lipid vesicles at 60 °C followed by slow cooling. The formation of this second bilayer also led to decreased fluorescence intensity from the first bilayer due to FRET. When both lipid bilayers phase-segregated, it was observed that the gel domains aligned between the two bilayers (Figure 5a, overlay), indicating coupling between the phase-segregation processes of both bilayers. Tayebi and collaborators [1] have previously reported the long-range alignment of phase-segregated lipid bilayer domains across hundreds of membrane lamellae. They postulated that such alignment originated from the surface tension associated with differences in the network of hydrogen-bonded water molecules at the hydrated interfaces between the domains and the surrounding phases. In our case, in addition to surface tension effects, the alignment could also be promoted by charges within the lipid bilayer, since the cationic and anionic lipids both partition preferentially into the liquid disordered phase. On the other hand, when the first cationic bilayer was not highly mobile, due to improper cleaning or cooling rate, it did not show visible phase segregation and the fluorescence intensity remained homogeneous (Figure 5b, green channel). Under this scenario, formation of the second anionic lipid bilayer led to phase segregation only on the second layer, where gel phase domains appeared dark (Figure 5b, red channel). In this case FRET between the two layers caused the green channel intensity from the first bilayer to show a complementary pattern to the phase segregation of the second bilayer (Figure 5b, overlaid image), resembling the results obtained from patterned SLB stacks where the first bilayer was homogeneous and the second one was phase segregating. These experimental results demonstrate that it is possible to pattern lipid bilayer stacks with heterogeneous lipid bilayers through the bilayer-by-bilayer and PSLO approach, and observe interactions between phase-segregating lipid mixtures across lipid lamellae. However, it must be noted that sample preparation and substrate cleanliness are key to obtaining highly-mobile lipid bilayers that enable such interactions.

## 3. Conclusions

In conclusion, we have demonstrated a simple bottom-up approach to pattern-stacked supported lipid bilayers using bilayer-by-bilayer deposition together with polymer stencil lift-off. Mobile SLBs were successfully stacked through electrostatic interactions between cationic and anionic lipids. This technique allowed patterning of both homogeneous and phase-segregated stacked SLB with controlled the number of bilayers and lipid compositions under aqueous conditions. We have demonstrated stacked SLB pattern formation with up to four bilayers consisting of all-homogeneous bilayers, phase-segregated on homogeneous bilayers, and phase-segregated on phase-segregated bilayers. The formation of patterned SLB stacks could be monitored through quantitative fluorescence microscopy. In addition, the fast fluorescence recovery in FRAP tests confirmed the good mobility of each stacked bilayer. For all patterned stacked SLBs, interactions between neighbouring bilayers could be observed through fluorescence resonance energy transfer between the dye molecules embedded in each bilayer. Furthermore, a study of lipid behaviour in neighbouring bilayers showed that phase-segregated domains align across the stack, provided the underlying membrane is fully mobile. We anticipate that the proposed stacked SLB patterning approach will be useful tool for biophysical studies of biomembrane and membrane-associated proteins. With the ability to control the number of bilayers in a stack, the multilamellar bilayer model could be an excellent platform for integrating transmembrane proteins, as it would prevent protein denaturation. Such a system can overcome the low fluidity, which arises in single SLBs due to the close proximity to the substrate, by increasing the distance between the top lipid bilayer and the substrate without modifying the lipid or introducing additional polymer layers. The ability to pattern stacked SLBs at the micron scale could also contribute to studying cell behaviour *in vitro*, because the patterns can be used to mimic cell microenvironments providing tunable substrate stiffness and adjustable biological components. The micropatterned multiple lipid bilayers are expected to be further applicable in building functional lipid-based devices for cell sensing and drug screening, where changes in bilayer stack thickness or order can be explored in a controlled environment.

## 4. Experimental Section

### 4.1. Materials

1,2-dioleoyl-sn-glycero-3-phosphocholine, chloride salt, (DOPC), 1,2-dioleoyl-3- trimethylammonium-propane, chloride salt, (DOTAP), 1,2-dimyristoyl-sn-glycero-3-phospho-L-serine, sodium salt, (DMPS) 1,2-dioleoyl-sn-glycero-3-phospho-L-serine, sodium salt (DOPS) and 1,2-distearoyl-sn-glycero-3-phosphocholine (DSPC) were purchased from Avanti Polar Lipids (Alabaster, AL). Lissamine Rhodamine B 1,2-dihexadecanoyl-sn-glycero-3-phosphoethanolamine, triethylammonium Salt (DHPE-LR) and Oregon Green 488 1,2-dihexadecanoyl-sn-glycero-3-phosphoethanolamine (DHPE-OG) were purchased from Life Technologies (Carlsbad, CA). Parylene-C was obtained from Specialty Coating Systems (Indianapolis, IN). Sodium hydroxide, 10× phosphate buffered saline (PBS) solution (which was diluted to 1× for buffer use), hydrochloric acid, hydrogen peroxide (30%), sulfuric acid (40%) and ammonium hydroxide (27%) were acquired from Caledon Laboratories (Georgetown, ON, Canada). L-ascorbic acid was obtained from Sigma-Aldrich (Saint Louis, MO, USA). Deionized water (18.2 MΩ·cm) was obtained from a Millipore Milli-Q Purification System (Millipore, Billerica, MA, USA).

### 4.2. Methods

#### 4.2.1. Microfabrication of Parylene Stencils

SiO_2_ (100 nm thermal oxide) on Si <100> wafers were cleaned in piranha solution (sulfuric acid:hydrogen peroxide 3:1 v/v) for 10 min, followed by two successive 5-min rinses in Milli-Q water, and then dried under a nitrogen stream. Then a ~800 nm thick Parylene film was deposited onto the clean wafers through chemical vapour deposition in a Labcoater 2000 Parylene deposition system (SCS, Indianapolis, IN, USA). S1808 positive photoresist (Shipley, Marlborough, MA, USA) was spin-coated on the Parylene-coated substrates at 2500 rpm for 30 s to obtain a nominal resist thickness of 1.1 μm, and then baked at 90 °C for 2 min. A chrome photo mask with 2–200 μm features (fabricated by Fineline Imaging Company, Colorado Springs, CO) was used in the patterning process. Photolithography was performed in soft-contact mode using a Karl Suss MJB3 contact aligner (UV wavelength 365 nm, Karl Suss, Garching, Germany), with exposure energy of 30 mJ. The samples were developed in Microposit 351 developer (Shipley, Malborough, MA, USA) for 25 s to remove the exposed photoresist, and then rinsed in Milli-Q water and dried under a nitrogen stream. Exposed regions of the Parylene film were reactive ion etched in an oxygen plasma chamber (Technics, Series 800, Arlington, TX, USA) for 9 min at 30 sccm O_2_, 100W. Residual photoresist was removed by washing successively with acetone, isopropanol, and Milli-Q water. To remove any remaining Parylene residue within the etched openings, the etched substrates were treated in a UV/O_3_ cleaner and further cleaned in an alkaline solution. Various UV/base conditions were tested, from which it was determined that one minute UV/O_3_ cleaning (10 mW/cm^2^, 254 nm), followed by 1 minute soaking in 100 mM sodium hydroxide solution provided the optimal surfaces for SLB formation. After this treatment, substrates were soaked in Milli-Q water for 1 min and dried under a nitrogen stream. Prior to use in lipid bilayer patterning, all the substrates were cleaned in a plasma chamber (Harrick, Ithaca, NY) for 60 s at high power using an air flow rate of 30 sccm.

#### 4.2.2. Preparation of Small Unilamellar Vesicle Solutions

Small unilamellar vesicle (SUV) solutions of different compositions were all prepared through the vesicle extrusion method. The lipids dissolved in chloroform were mixed at the desired ratios to ensure a homogeneous mixture of the lipids. Afterwards, the chloroform was removed by rotatory evaporation using a stream of nitrogen to produce dry lipid films, and then any remaining solvent was evaporated under vacuum overnight. The lipid films were hydrated with the required volume of 1× PBS until the lipid films completely resuspended forming a cloudy solution containing large multilamellar vesicles (LMV) of various sizes. The LMV solutions were pre-filtered through a 0.45 μm pore polyethersulfone filtering membrane to remove impurities and reduce the vesicle size. The solutions were then extruded 10 times through a 100 nm pore polycarbonate membrane filter (Whatman, Maidstone, UK) using a mini-extruder (Avanti Polar Lipids, Alabaster, AL, USA). This resulted in clear solution containing SUVs of approximate 100 nm diameter. Lipid compositions are expressed as molar ratios. The solutions used for these experiments were 1 mM DOPC:DOTAP:DHPE-OG (90:10:0.1), 1 mM DOPC:DMPS:DHPE-LR (90:10:0.1), 1mM DOPC:DSPC:DOPS:DHPE-LR (80:10:10:0.1) and 1 mM DOPC:DSPC:DOTAP:DHPE-OG (80:10:10:0.1).

#### 4.2.3. Formation of Stacked Supported Lipid Bilayers

Supported lipid bilayers were formed by applying the SUV solution onto the substrate surface. Prior to SLB formation, the wafers with Parylene stencils on top were air plasma cleaned for 1 minute using the Expanded Plasma Cleaner a PlasmaFlo (Harrick, Ithaca, NY, USA) to remove adsorbed impurities and increase the number of silanol groups providing a more negatively charged surface for better electrostatic interactions with the charged lipids. Afterwards, 0.2 mM lipid SUV solution of interest was deposited on the micropatterned surfaces and incubated under appropriate conditions until a uniform SLB formed. The incubation temperatures were set higher than the transition temperatures of the lipids in the solution to ensure all lipids are at the liquid phase. The homogenous SLBs were prepared by incubating the appropriate SUV solutions on the patterned substrates for 60 min at 30 °C, while phase-segregated SLBs were prepared by incubating the appropriate SUV solutions for 30 min at 60 °C. In order to remove excess lipid vesicles, the surface was carefully washed three times with 1× PBS (pH = 6) supplemented with 5 mM ascorbic acid, followed by another three washes with 1× PBS (pH = 9) supplemented with 5 mM ascorbic acid. Both the ascorbic acid and PBS buffer at pH 9 were used to reduce the rate at which the DHPE-OG fluorophore bleaches for better imaging and analysis with the fluorescence microscope. The formed SLBs were then allowed to cool to room temperature. For both the multi-layer phase-segregated and homogeneous SLB, the Parylene stencil was removed after the formation of all the layers and right before the characterization was conducted.

#### 4.2.4. Mobility and Intensity Characterization by Fluorescence Microscopy

The distinctive bilayers were imaged using a Nikon Eclipse LV100N POL epifluorescence microscope (Nikon Instruments, Mississauga, ON, USA) equipped with excitation and emission filters for Oregon Green or Lissamine Rhodamine dyes, and an UMPLNFL 20×/0.5NA objective. Images were acquired with a Retiga 2000R cooled CCD camera (QImaging, Surrey, BC, Canada) and recorded with software NIS-Elements AR (Nikon, Tokyo, Japan).

Fluorescence recovery after photobleaching (FRAP) was used for SLB mobility tests in all the experiments. This technique began by saving an image of the sample before photobleaching. The light source was then briefly focused onto a small area of the SLB. The fluorophores in this region received high intensity illumination and bleached quickly, leaving a dark region behind. The sample fluorescence was monitored at subsequent time intervals. As diffusion proceeded, the bright molecules diffused into the bleached region. The fluorescence could recover after a while, demonstrating that the lipids retained their fluidity on the substrates.

The fluorescence intensity for each of the four layers in the multilamellar homogeneous SLB was measured and compared to prove the presence of each lipid bilayer. The intensity profiles were obtained for five different micropatterened areas on each layer using the NIS Elements software, and the average intensity was calculated for comparison. The parameters used for acquiring pictures of green fluorophore (DHPE-OG) labeled layers were 3.0 gain and 100 ms exposure time; while for the bilayers containing the red fluorophore (DHPE-LR), the parameters were set to 5.0 gain and 100 ms exposure time. However for the phase-segregated layer labeled with LR dye, the exposure time was 50 ms.

## Supplementary Files

Supplementary File 1
