# Supplementary material for: Multi-Stacked Supported Lipid Bilayer Micropatterning through Polymer Stencil Lift-Off
Source: Membranes (Basel). 2015 Aug 28;5(3):385–98. doi: 10.3390/membranes5030385 (PMC4584287; doi:10.3390/membranes5030385)
Supplement: Supplementary File 1 [file membranes-05-00385-s001.pdf]

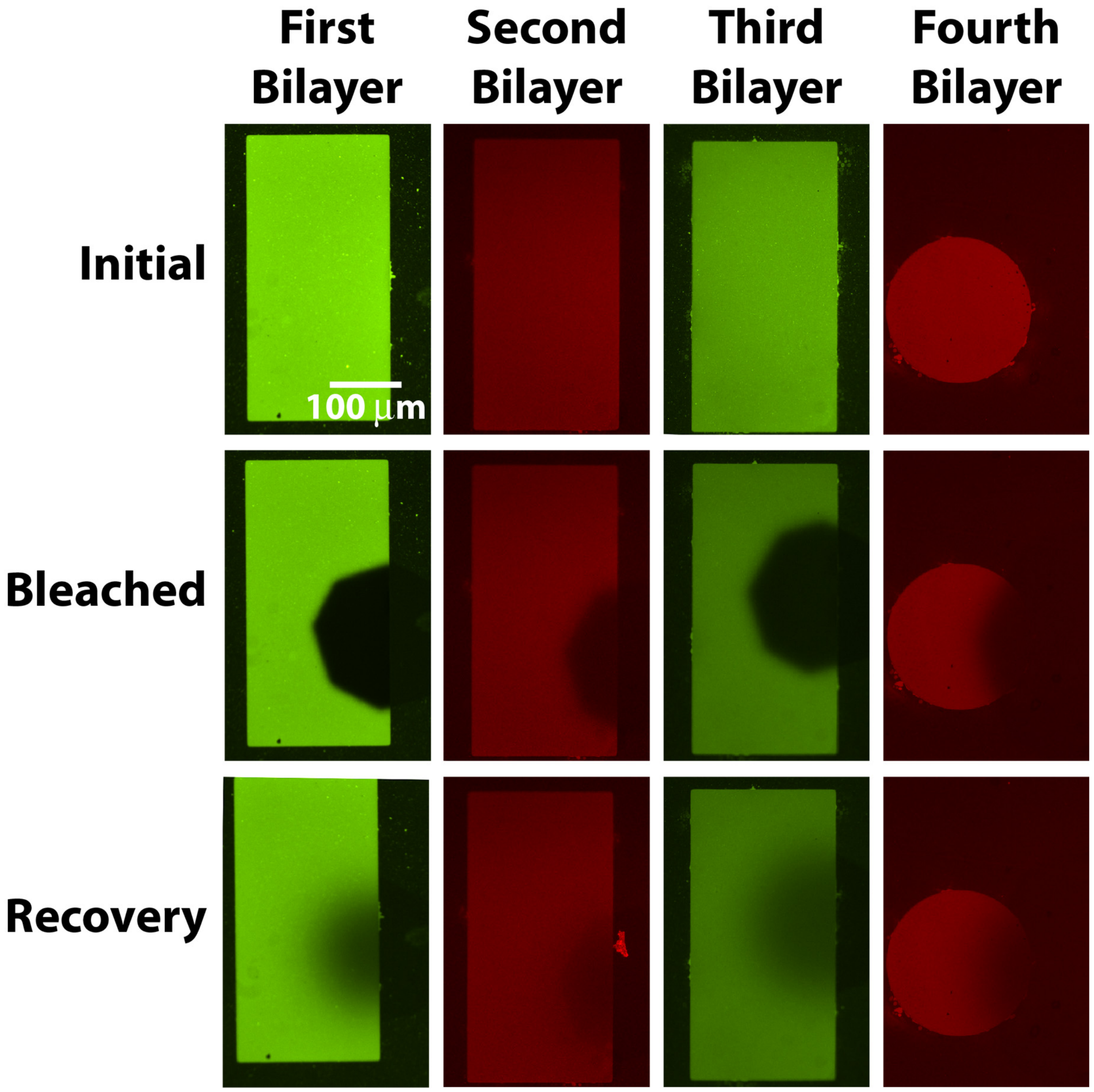

Figure S1. Fluorescence recovery after photobleaching (FRAP) test for four homogeneous bilayer stack SLB micropatterns. Epifluoresence images of each layer were taken before photobleaching, after photobleaching, and after partial fluorescence recovery. All images were acquired at the same magnification.

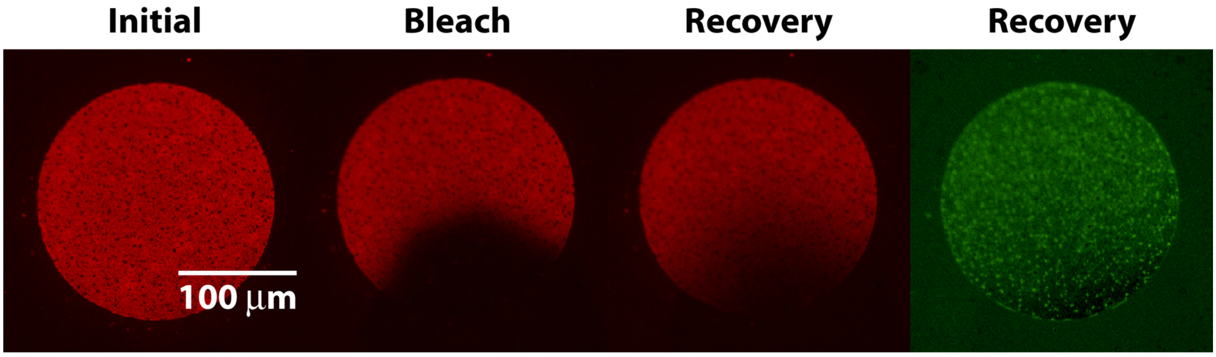

Figure S2. Epifluorescence images of fluorescence recovery after photobleaching (FRAP) test for a two-bilayer SLB stack composed of a phase-segregated bilayer (red) deposited on a homogeneous bilayer (green). Epifluoresence images of each layer were taken before photobleaching, after photobleaching, and after partial fluorescence recovery.
